# Supplementary material for: Mapping the evolution of fertility support policies in China: A content and instrumental analysis
Source: PLoS One. 2025 Oct 9;20(10):e0332137. doi: 10.1371/journal.pone.0332137 (PMC12510515; doi:10.1371/journal.pone.0332137)
Supplement: S1 Appendix — (ZIP) [file pone.0332137.s001.zip › S1 Appendix. 226 original policy documents/128-卫生部办公厅关于印发《乡村医生在岗培训基本要求》的通知(FBM-CLI-4-51351).doc]

卫生部办公厅关于印发《乡村医生在岗培训基本要求》的通知

**发布部门：** [卫生部(已撤销)](https://www.pkulaw.com/law/chl?Aggs.IssueDepartment=60222) [机构沿革](javascript:void(0))

**发文字号：**卫办科教函〔2004〕4号

**发布日期：**2004.01.12

**实施日期：**2004.01.12

**时效性：** [现行有效](https://www.pkulaw.com/law/chl?Aggs.TimelinessDic=01)

**效力级别：** [部门规范性文件](https://www.pkulaw.com/law/chl?Aggs.EffectivenessDic=XE0302)

**法规类别：** [卫生机构与人员](https://www.pkulaw.com/law/chl?Aggs.Category=09602)

　　　　　　　　　　　　　卫生部办公厅关于印发
　　　　　　　　　《乡村医生在岗培训基本要求》的通知
　　　　　　　　　　　　（卫办科教函[2004]4号）

各省、自治区、直辖市卫生厅局、乡村医生培训中心，新疆生产建设兵团卫生局，中国乡村医生培训中心：
　　为贯彻《[乡村医生从业管理条例](https://www.pkulaw.com/chl/1b710d20ffca23b2bdfb.html)》，切实加强乡村医生培训工作，规范管理，逐步完善乡村医生终身学习制度，我部在调查研究的基础上，组织制定了《乡村医生在岗培训基本要求》，现印发给你们，请遵照执行。执行中有何意见和建议，请及时反馈我部。
　　附件：乡村医生在岗培训基本要求
　　　　　　　　　　　　　　　　　　　　　　　　　二00四年一月十二日

附件：　　　　　　 乡村医生在岗培训基本要求

　　**一、**素质要求
　　要求掌握内容：
　　（一）医德医风
　　（二）医学伦理
　　（三）法律法规
　　（四）方针政策

　　**二、**业务要求
　　（一）农村初级卫生保健
　　要求掌握内容：
　　1．计划免疫
　　2．传染病系统管理的内容和方法
　　3．流行病学个案调查及方法
　　4．常用消毒剂、杀虫剂的药理作用及使用方法与配制
　　5．“两管五改”的基本知识和应用技术
　　6．生存环境与健康的基本内容
　　7．健康、健康传播、健康心理、健康行为
　　8．卫生防病知识宣传和卫生行为科学指导
　　9．常用农村基层卫生统计
　　10．村卫生室的规范化管理
　　（二）全科医学与社区卫生服务
　　要求掌握内容：
　　1．全科医学、全科医疗、全科医生的概念
　　2．全科医疗的基本内容
　　3．社区卫生服务的功能及基本内容
　　（三）妇女保健、儿童保健以及计划生育技术指导
　　要求掌握内容：
　　1．妇科常见病的一般性诊断、治疗和预防原则
　　2．优生优育、避孕、节育措施和方法
　　3．围产期保健及科学接生
　　4．性知识教育（含常见性病的预防）
　　5．更年期综合征的防治
　　6．妇女、儿童保健的基本知识和内容
　　（四）中医、中药基本知识
　　要求掌握内容：
　　1．中医基础
　　2．常用中药（成药20种以上、草药100种以上）
　　3．常用方剂（50个以上）
　　4．常见证（20个以上）
　　5．常见病（20种以上）
　　6．针灸、推拿及按摩的基本知识和基本操作
　　（五）医学基础
　　1．解剖学
　　（1）运动系统
　　要求掌握内容：骨　关节　骨骼肌
　　（2）消化系统
　　要求掌握内容：消化管　消化腺　腹膜
　　（3）呼吸系统
　　要求掌握内容：气管、支气管　肺和胸膜
　　（4）泌尿系统
　　要求掌握内容：肾　输尿管　膀胱　尿道
　　（5）生殖系统
　　要求掌握内容：男性生殖系统的组成　精子排出的途径
　　女性内、外生殖器　骨盆
　　（6）脉管系统
　　要求掌握内容：心　动脉　静脉　淋巴
　　（7）周围神经系统
　　要求掌握内容：脊神经　脑神经　植物性神经
　　（8）中枢神经系统
　　要求掌握内容：脊髓的主要功能　脑
　　（9）内分泌腺
　　要求掌握内容：人体的内分泌腺名称及位置
　　2．生理学
　　（1）绪论
　　要求掌握内容：生命的基本特征　机体的内环境　机体功能活动的调节
　　（2）细胞的基本功能
　　要求掌握内容：细胞膜的物质转运功能
　　（3）血液及血液循环
　　要求掌握内容：血液的组成和血量　血细胞的基本功能　血型　循环系统的基本功能
　　（4）呼吸
　　要求掌握内容：肺的通气功能　肺内气体交换　气体在血液中的运输
　　（5）消化与吸收
　　要求掌握内容：消化道各段的消化功能　吸收　消化器官活动的调节
　　（6）体温
　　要求掌握内容：体温的概念、正常值及生理变异　机体的产热和散热
　　（7）肾脏的排泄功能
　　要求掌握内容：概述　尿的生成过程　影响尿生成因素
　　（8）神经系统
　　要求掌握内容：交感神经和副交感神经的主要功能
　　（9）感觉器官
　　要求掌握内容：视觉器官和听觉器官
　　（10）内分泌
　　要求掌握内容：概述　甲状腺　肾上腺
　　（11）生殖
　　要求掌握内容：妇女一生各阶段的生理特点　卵巢的内分泌功能　月经及月经周期变化
　　3．药理学
　　（1）总论
　　要求掌握内容：药效学　药动力学　影响药物作用的因素
　　（2）传出神经系统药
　　要求掌握内容：胆碱受体激动药与胆碱脂酶抑制药　胆碱受体阻断药　肾上腺素受体激动药　肾上腺素受体阻断药
　　（3）局部麻醉药
　　要求掌握内容：普鲁卡因　利多卡因
　　（4）中枢神经系统药
　　要求掌握内容：镇静催眠药　抗精神失常药　镇痛药　解热镇痛抗炎药　中枢兴奋药
　　（5）心血管系统药
　　要求掌握内容：抗高血压药　抗心绞痛药　抗心律失常药　抗慢性心功能不全药
　　（6）利尿与脱水药
　　要求掌握内容：利尿药　甘露醇
　　（7）抗过敏药
　　要求掌握内容：H1受体阻断药　钙剂
　　（8）呼吸系统药
　　要求掌握内容：镇咳药　怯痰药　平喘药
　　（9）消化系统药
　　要求掌握内容：抗消化性溃疡药　泻药
　　（10）子宫兴奋药
　　要求掌握内容：催产素　麦角新碱
　　（11）血液与造血系统药
　　要求掌握内容：抗贫血药　影响血凝过程药
　　（12）激素类药
　　要求掌握内容：糖皮质激素的正确使用方法及不良反应和注意事项 胰岛素及口服降血糖药
　　（13）抗微生物药
　　要求掌握内容：抗生素　人工合成抗菌药　抗结核药
　　（14）抗寄生虫药
　　要求掌握内容：抗疟药　抗肠蠕虫药
　　（六）临床实践
　　1．医学概论
　　2．常见症状及体征、一般体格检查
　　要求掌握内容：常见症状　常见体征　常用体格检查的基本方法及标准操作
　　3.急救
　　要求掌握内容：各种常见急救技术（内、外、妇、儿、五官等科）
　　4．呼吸系统（含小儿）常见疾病的预防、诊断和治疗、急救原则以及转送上级医院注意事项和办法
　　要求掌握内容：急性上呼吸道感染 急、慢性支气管炎
　　阻塞性肺气肿　支气管哮喘　支气管扩张症　肺炎球菌肺炎　肺结核
　　5．循环系统常见疾病的预防、诊断和治疗、急救原则以及转送上级医院注意事项和办法
　　要求掌握内容：休克　高血压　心绞痛
　　6．消化系统常见疾病（含外科、儿科）的预防、诊断和治疗、急救原则以及转送上级医院注意事项和办法
　　要求掌握内容：常见症状　急、慢性胃炎　消化性溃疡
　　外科急腹症的特点及体征 婴幼儿腹泻
　　7．泌尿系统（含小儿）常见疾病的预防、诊断和治疗原则以及转送上级医院注意事项和办法
　　要求掌握内容：肾小球疾病概述　急、慢性肾小球肾炎
　　肾盂肾炎　尿路感染
　　8．造血系统常见疾病的预防、诊断和治疗原则以及转送上级医院注意事项和办法
　　要求掌握内容：贫血疾病概述 缺铁性贫血
　　9．内分泌及代谢（含小儿）常见疾病的预防、诊断和治疗原则以及转送上级医院注意事项和办法
　　要求掌握内容：内分泌及代谢（含小儿）常见疾病概述
　　单纯性甲状腺肿　甲状腺机能亢进症
　　糖尿病
　　10．风湿性常见疾病的预防、诊断和治疗原则以及转送上级医院注意事项和办法
　　要求掌握内容：风湿性常见疾病特点 类风湿关节炎
　　11．理化因素所致常见疾病的预防、诊断和治疗、急救原则以及转送上级医院注意事项和办法。
　　要求掌握内容：急性中毒概论　有机磷农药中毒　急性一氧化碳中毒　中暑　溺水
　　12．神经及精神系统常见疾病的预防、诊断和治疗原则；以及转送上级医院注意事项和办法
　　要求掌握内容：脑出血　脑栓塞　三叉神经痛　癫痫
　　精神疾病常见症状及表现　神经衰弱　癔症
　　13．临床常用诊疗技术及基本（标准）操作（含内、外、妇、儿）
　　要求掌握内容：常用消毒及灭菌技术　体温、脉搏、呼吸、血压的标准测量　药物过敏试验法　药物过敏的急救原则　各种注射法　洗胃法　导尿与膀胱冲洗法　酒精及温水擦浴　冷、热敷
　　（七）医学心理学
　　要求掌握内容：
　　1．医学心理学概述
　　2．心理卫生
　　3．病人心理
　　4．医患关系

　　**三、**管理要求
　　乡村医生在岗培训工作实行登记考核制度。乡村医生参加在岗培训活动的名称、形式、学时、日期及考核结果等情况要进行登记。乡村医生每两年参加集中培训时间不得少于100学时。

© 北大法宝：（[www.pkulaw.com](https://www.pkulaw.com/)) 专业提供法律信息、法学知识和法律软件领域各类解决方案。北大法宝为您提供丰富的参考资料，正式引用法规条文时请与标准文本核对。
欢迎查看所有[产品和服务](https://www.pkulaw.com/helps/2.html)。法宝快讯：[如何快速找到您需要的检索结果？](https://www.pkulaw.com/helps/70.html)[法宝](https://www.pkulaw.com/helps/69.html)V6有何新特色？

扫描二维码阅读原文

**原文链接：**<https://www.pkulaw.com/chl/ba7cbdb6d6c8a63abdfb.html>
